# Replicative senescence dictates the emergence of disease-associated microglia and contributes to Aβ pathology

**DOI:** 10.1016/j.celrep.2021.109228

**Published:** 2021-06-08

**Authors:** Yanling Hu, Gemma L. Fryatt, Mohammadmersad Ghorbani, Juliane Obst, David A. Menassa, Maria Martin-Estebane, Tim A.O. Muntslag, Adrian Olmos-Alonso, Monica Guerrero-Carrasco, Daniel Thomas, Mark S. Cragg, Diego Gomez-Nicola

**Affiliations:** 1 School of Biological Sciences, University of Southampton, Southampton General Hospital, Southampton, UK; 2 Antibody and Vaccine Group, Centre for Cancer Immunology, Faculty of Medicine, University of Southampton, Southampton General Hospital, Southampton, UK

**Keywords:** disease-associated microglia (DAM), CSF1R, Alzheimer's disease, APP/PS1

## Abstract

The sustained proliferation of microglia is a key hallmark of Alzheimer’s disease (AD), accelerating its progression. Here, we aim to understand the long-term impact of the early and prolonged microglial proliferation observed in AD, hypothesizing that extensive and repeated cycling would engender a distinct transcriptional and phenotypic trajectory. We show that the early and sustained microglial proliferation seen in an AD-like model promotes replicative senescence, characterized by increased βgal activity, a senescence-associated transcriptional signature, and telomere shortening, correlating with the appearance of disease-associated microglia (DAM) and senescent microglial profiles in human post-mortem AD cases. The prevention of early microglial proliferation hinders the development of senescence and DAM, impairing the accumulation of Aβ, as well as associated neuritic and synaptic damage. Overall, our results indicate that excessive microglial proliferation leads to the generation of senescent DAM, which contributes to early Aβ pathology in AD.

## Highlights

• In Alzheimer’s-like pathology, a fraction of microglia undergo replicative senescence
• Disease-associated microglia (DAM) display several features of senescence
• Prevention of proliferation impairs the development of microglial senescence and DAMs
• Prevention of microglial senescence leads to reduced amyloidosis and synaptic damage

## Graphical abstract

**Figure:**
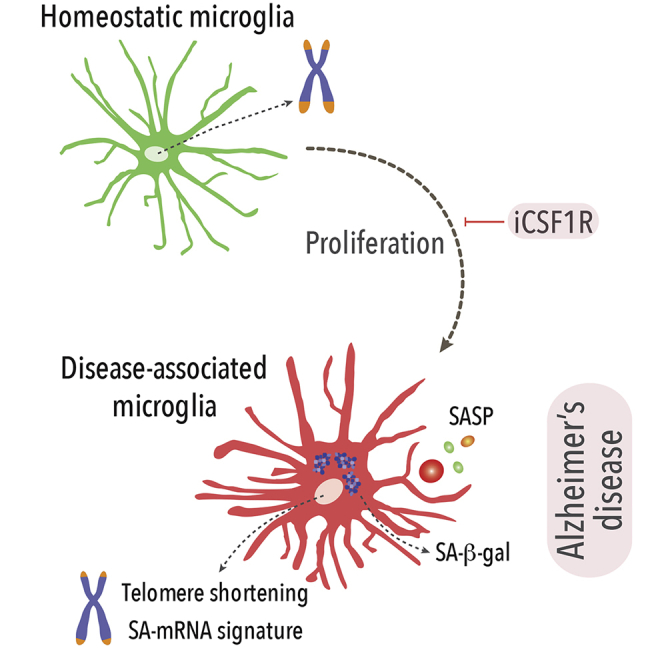

## Teaser

Hu et al. show that microglia undergo replicative senescence in a model of Alzheimer’s-like pathology. Senescence is enriched in disease-associated microglia and is caused by the accumulation of proliferative cycles. Prevention of microglial senescence leads to diminished pathology, highlighting a link of microglia with the early stages of Alzheimer’s disease.

## Introduction

Microglia, the main resident macrophages of the brain, originate from yolk-sac progenitors that invade the brain primordium during early embryonic development (Ginhoux et al., 2010). These founders undergo several cycles of proliferation during embryonic and early postnatal development to achieve the numbers and distribution observed in the adult brain (Alliot et al., 1999; Nikodemova et al., 2015; Askew and Gomez-Nicola, 2018). In the adult steady state, the microglial population undergoes several rounds of renewal, through a slow turnover mechanism of proliferation being temporally and spatially coupled to intrinsic apoptosis (Askew et al., 2017).

The re-activation of microglial proliferative programs is the earliest response to pre-pathological events in chronic neurodegenerative diseases, with microglial proliferation increased in Alzheimer’s disease (AD) (Olmos-Alonso et al., 2016; Simon et al., 2019). Microglia have a very rapid proliferative response to the incipient accumulation of Aβ (Condello et al., 2015), during the onset of tau pathology (Mancuso et al., 2019) and in several other related models of neurodegeneration (Martínez-Muriana et al., 2016; Clayton et al., 2017). This rapid response is observed by the fast transition to a proliferative transcriptional state triggered shortly after disease onset in the CK-p25 model of neurodegeneration (Mathys et al., 2017). We and others have demonstrated that the proliferation of microglia is a central contributor to disease progression. The inhibition of microglial proliferation, using CSF1R inhibitors, ameliorates amyloid (Olmos-Alonso et al., 2016; Spangenberg et al., 2016; Dagher et al., 2015) and tau pathology (Mancuso et al., 2019) and has emerged as a promising target for clinical investigation. Interestingly, microglial cells entering early proliferation in disease later undergo phenotypic specification into a disease-associated microglia (DAM) (Mathys et al., 2017) by unknown mechanisms. DAM represent a key microglial subpopulation present across several brain disorders and is dependent on triggering receptor expressed on myeloid cells 2-apolipoprotein E (TREM2-APOE) signaling (Krasemann et al., 2017; Keren-Shaul et al., 2017). However, the specific mechanisms by which microglial proliferation evokes the DAM phenotype and how this is related to synaptic and neuronal degeneration has yet to be defined.

Integrating our knowledge of microglial population dynamics renders an interesting hypothesis. When combined, the cycling events accumulated in microglia from development to disease would put these cells on a trajectory toward cellular senescence. Replicative senescence, the loss of mitotic potential accompanied by significant telomere shortening, occurs once a cell has undergone ∼50 replications, the so-called Hayflick limit (Hayflick, 1965). Thus, we hypothesized that the developmental setup of the population, combined with microglial turnover, would pre-condition these cells to undergo replicative senescence when challenged with additional proliferative events (i.e., as a consequence of brain pathology). Some reports suggest that microglia show telomere shortening and decreased telomerase activity in both aging (Flanary and Streit, 2004) and end-stage AD (Flanary et al., 2007). However, to date, no formal evidence has been provided supporting the idea that these progressive changes in the dynamics of microglia are driving the shift of the microglial response from beneficial to detrimental and therefore contributing to the initiation of AD.

Here, we provide evidence that microglia undergo replicative senescence in a model of AD-like pathology and in human AD. We demonstrate that DAM display a senescence-associated profile and that the mechanism for phenotypic specification is dependent on proliferation. Our data support that the early generation of senescent microglia contributes to the subsequent onset and progression of amyloidosis, as well as the associated neuritic damage that is observed in the early stages of AD.

## Results

### DAM appear shortly after the onset of plaque pathology, in line with a progressive proliferation of microglia

**Figure 1:**
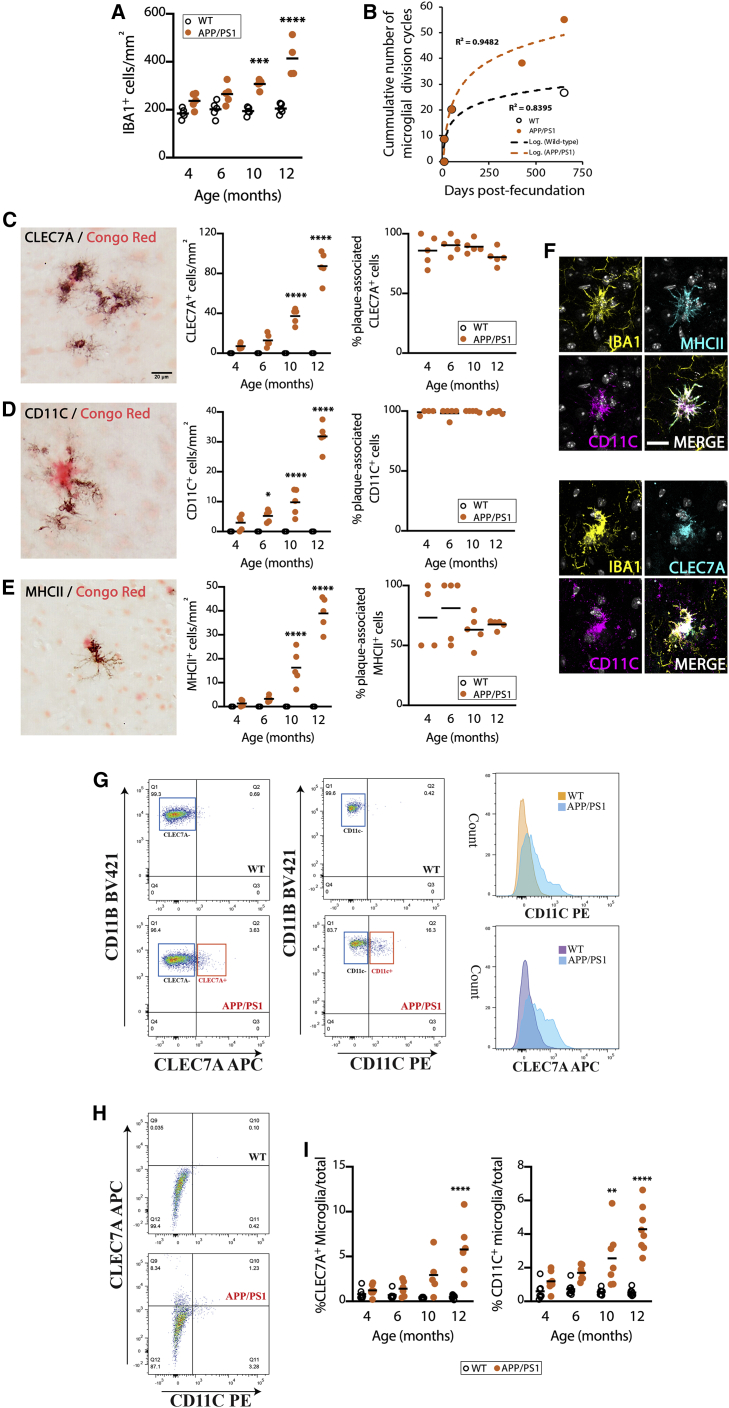
Dynamics and phenotypic specification of DAM in APP/PS1 mice (A) Time course of the microglial (IBA1^+^) density in APP/PS1 mice and age-matched controls, analyzed by IHC. (B) Representation of the cumulative number of microglial division cycles from early colonization of the brain primordium to aging (20 months of age) in WT and APP/PS1 mice, based on retrospective analysis of published data (Askew et al., 2017; Olmos-Alonso et al., 2016; Alliot et al., 1999; Nikodemova et al., 2015). (C–E) Time course of the density and plaque association (%) of DAM, identified as CLEC7A^+^ (C), CD11C^+^ (D), or MHCII^+^ (E) cells, in APP/PS1 mice and WT littermate controls, analyzed by immunohistochemistry (IHC) (black). Aβ plaques labeled with Congo Red (C)–(E). (F) Confocal imaging of the co-localization of MHCII, CD11C, and CLECL7A in plaque-associated microglia (IBA1^+^) in APP/PS1 mice. Nuclei stained with DAPI, shown in grayscale. (G–I) Flow cytometry analysis of the expression of markers of DAM (CLEC7A, CD11C) in microglia (CD11B^+^CSF1R^+^) in APP/PS1 and WT controls. Immunonegative versus immunopositive gates for CD11C represented as cell count in a histogram plot. Co-expression pattern of CLEC7A and CD11C in microglia (H), analyzed as in (G). Quantification of the frequency of CLEC7A^+^ or CD11C^+^ microglia shown in (I). IHC data collected from average of parietal, auditory, and entorhinal cortex. Flow performed in samples from cerebral cortex. Scale bars in (C)–(F), 20 μm, shown in (C) and (F). Data shown in (A), (C)–(E), and (I) represented as means SEMs. N = 4–5 (A–F), N = 6–8 (G–I). Statistical differences: ^∗^p < 0.05, ^∗∗^p < 0.01, ^∗∗∗^p < 0.001, and ^∗∗∗∗^p < 0.0001 versus age-matched controls. Data were analyzed with a 2-way ANOVA and post hoc Tukey tests.

In the APP/PS1 model of AD-like pathology, IBA1^+^ cells (microglia) increase in number from the onset of plaque pathology by 4 months of age, with pronounced changes by 12 months of age (Figure 1A). We integrated these data with previously published datasets analyzing microglial densities from early development to aging (Alliot et al., 1999; Nikodemova et al., 2015; Askew et al., 2017; Olmos-Alonso et al., 2016) to calculate the number of cycles required to expand the microglia population from a limited starting number of progenitors (Figure 1B). The addition of the initial number of cycles required to provide and self-renew the adult density (Askew et al., 2017) to the additional proliferative cycles in APP/PS1 mice (Olmos-Alonso et al., 2016) places microglia in proximity to the threshold of 50 replicative cycles, the Hayflick limit (Hayflick, 1965) (Figure 1B). The appearance of DAM is also an early event, with CLEC7A^+^, CD11C^+^, and MHCII^+^ cells (all markers of DAM; Keren-Shaul et al., 2017) seen in close association with Aβ plaques from early time points (Figures 1C–1F). Notably, these markers are not expressed in WT microglia (Figure S1). Using flow cytometry, microglia can also be characterized as CSF1R^+^CD11B^+^ (Figure S2), with a subpopulation observed to progressively acquire key DAM markers (Figures 1G–1I), with the expression of these markers displaying high correlation (CD11C versus CLEC7A, R^2^ = 0.92, p < 0.0001).

### DAM display a phenotype characteristic of senescent cells, including telomere shortening

**Figure 2:**
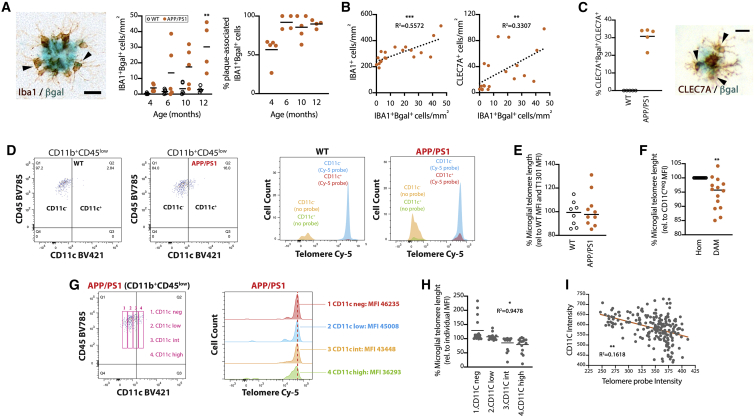
Microglial senescence in APP/PS1 mice (A) Time course of the density of βgal^+^ (blue) microglia (IBA1^+^; brown) in APP/PS1 mice and age-matched controls, analyzed by IHC. Representative βgal^+^IBA1^+^ cells identified with an arrowhead. Time course of the plaque association (%) of βgal^+^IBA1^+^ microglia, in APP/PS1 mice. (B) Correlation of the density of senescent microglia (βgal^+^IBA1^+^) with the total density of microglia (IBA1^+^) or the density of DAM (CLEC7A^+^). R^2^ of linear regression analysis shown in plots. (C) Expression of βgal in CLEC7A^+^ cells in APP/PS1 mice and age-matched controls, analyzed by IHC and shown as percentage of total CLEC7A^+^ cells. Representative βgal^+^CLEC7A^+^ cells identified with an arrowhead. (D–F) Flow-FISH analysis of the telomere length (Cy-5 probe) in microglia (CD11B^+^CD45^low^), identifying DAM by CD11C^+^ expression in APP/PS1 mice and WT littermates. Immunonegative versus immunopositive gates for the telomere probe in WT and APP/PS1 microglia, as well as negative controls, represented as cell count in a histogram plot. Relative telomere length (percentage corrected to internal T1301 signal and relative to WT) in total microglia (E) and in homeostatic (Hom; CD11C^−^) versus DAM (CD11C^+^) in APP/PS1 mice (F). (G–I) Analysis of the relative telomere length in specific subpopulations of microglia (CD11B^+^CD45^low^), identified by CD11C expression as negative (CD11C^neg^), low (CD11C^low^), intermediate (CD11C^int^), and high (CD11C^high^). (G) Representative gates and their cell count for the telomere probe shown in a histogram plot. (H) Quantification of the relative telomere length (percentage relative to individual average CD11C MFI) in 4 CD11C gates in APP/PS1 mice. (I) Correlation of CD11C intensity versus telomere probe intensity in a representative APP/PS1 mouse. R^2^ of the correlation analyses shown in (H and I). IHC data collected from average of parietal, auditory, and entorhinal cortex. Scale bars in (A and C), 20 μm. Flow cytometry performed in samples from cerebral cortex. Data represented as means ± SEMs. N = 4–5 (A–C), N = 8–14 (D–I). Statistical differences: ^∗∗^p < 0.01 versus age-matched control (A); ^∗^p < 0.05, ^∗∗^p < 0.01, and ^∗∗∗^p < 0.001 correlation analysis, linear regression (B, H, and I); ^∗∗^p < 0.01 versus homeostatic microglia (Hom; F). Data were analyzed with a 2-way ANOVA and post hoc Tukey tests (A) and unpaired t test (E and F).

We studied the increase of senescence-associated β-galactosidase activity (SA-βgal) in microglia in APP/PS1 mice, as a key feature of cells undergoing senescence (Lee et al., 2006). A fraction of microglia proximal to Aβ plaques displayed increased cytoplasmic βgal activity, representing 4.6% ± 0.9% (mean ± SEM) of the total population (Figure 2A). βgal^+^ microglia remained almost exclusively associated with Aβ plaques from 6 months of age (Figure 2A). At an earlier stage of the pathology, at 4 months of age, we did not observe plaque association of βgal^+^IBA1^+^ microglia, likely due to the suboptimal identification of nascent plaques at this stage (Figure 2A). When combining all ages, the density of senescent microglia (IBA1^+^βgal^+^) correlated with the density of total microglia (IBA1^+^) and DAM (CLEC7A^+^) (Figure 2B). Analyzing the cytoplasmic βgal activity in DAM, we found that 30% of CLEC7A^+^ cells were positive for βgal (Figure 2C). This indicates that a significant proportion of DAM display increased βgal activity.

We next set out to analyze the relative telomere length of microglia, as a direct measure of replicative senescence. Detecting binding of a telomere-specific probe by flow cytometry-fluorescence *in situ* hybridization (Flow-FISH) allows for the analysis of telomere length, as observed when combining cells with known telomere lengths like Jurkat (short) and T1301 (long) (Figure S3). Using T1301 as an internal control, we implemented a flow cytometric method for quantifying the relative telomere length of microglia, characterized as CD11B^+^CD45^low^ cells (Figures 2D and S4). The relative telomere length of the global population of microglia in APP/PS1 mice was not statistically different from that of wild-type (WT) mice (Figures 2D and 2E). However, when comparing DAM (CD11C^+^) to homeostatic microglia (CD11C^−^), we observed a significant telomere shortening in DAM (Figure 2F). Considering that the acquisition of the DAM phenotype is characterized by the progressively increasing expression of CD11C (Figure 1H), we gated microglia by 4 levels of CD11C expression (negative, low, intermediate, high) in APP/PS1 mice, observing a progressive reduction in telomere length in microglia expressing progressively higher CD11C (Figures 2G and 2H). The expression of CD11C inversely correlated with the relative telomere length, at the CD11C cell subpopulation level (Figure 2H) and between individual cells, considering CD11C as a continuous variable (Figure 2I).

### DAM display a transcriptional signature characteristic of senescent cells

**Figure 3:**
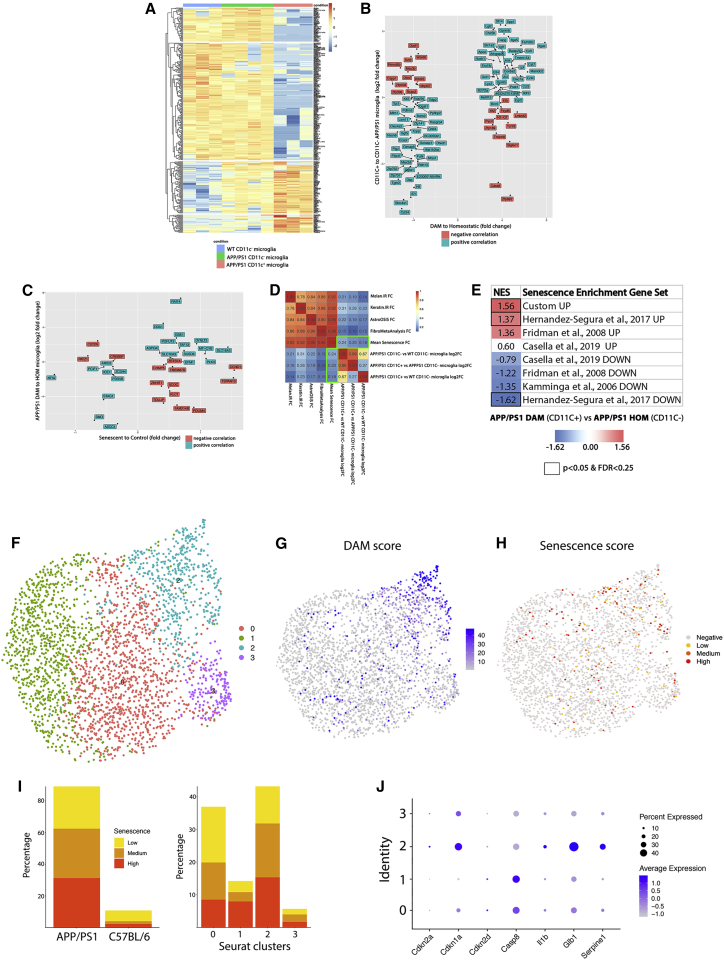
DAM display a senescent transcriptional signature (A) Heatmap representation of the log2 fold expression of genes from the DAM signature (Keren-Shaul et al., 2017) in WT CD11C^−^ microglia (blue), APP/PS1 CD11C^−^ microglia (green), and APP/PS1 CD11C^+^ microglia (red), using the pheatmap package. (B) Correlation analysis of the top 100 genes with highest and lowest fold change from Keren-Shaul et al. (2017) alongside the log2 fold change comparison of CD11C^+^ versus CD11C^−^ microglia from APP/PS1 mice, using the ggplot2 package. (C) Correlation analysis of the fold change of genes from the core senescence signature (Hernandez-Segura et al., 2017), with low read genes filtered out, alongside the log2 fold change comparison of APP/PS1 CD11C^+^ microglia versus WT CD11C^−^ microglia, using pheatmap and corrplot packages. (D) Correlation analysis of the genes from the senescence-associated signature of melanocytes, keratinocytes, astrocytes, fibroblasts, and core senescence signature (boxed in green) (Hernandez-Segura et al., 2017), with low read genes filtered out, with microglia from APP/PS1 and WT mice, using the corrplot package. (E) Gene set enrichment analysis (Mootha et al., 2003; Subramanian et al., 2005) of signatures upregulated or downregulated in senescence cells (Hernandez-Segura et al., 2017; Fridman and Tainsky, 2008; Casella et al., 2019; Kamminga et al., 2006), as well as a custom signature of genes highly associated with senescent cells (see Results section). Normalized enrichment score (NES) shown for the comparison of DAM (CD11C^+^) versus homeostatic microglia (CD11C^−^) from APP/PS1 mice. NES reaching a p < 0.05 and FDR < 0.25 highlighted by a squared NES. (F–J) Analysis of the single-cell dataset from Van Hove et al. (2019). (F) Uniform manifold approximation and projection (UMAP) plot of the microglial clusters identified from the original dataset after subsetting based on enriched expression of *Sall1*, *Gpr34*, *Tmem119*, *Hexb*, *P2ry12*, and *Cx3cr1* from the whole brains of 16-month-old APP/PS1 and WT mice. (G) Feature plot of the DAM signature (*Cst7*, *Csf1*, *Lpl*, *Apoe*, *Spp1*, *Cd74*, *Itgax*), identifying cluster 2 as DAM. Further annotation of the clusters (see Figure S5) identified cluster 1 as homeostatic microglia in C57BL/6 mice, cluster 0 as homeostatic microglia in APP/PS1 mice, cluster 3 as white matter microglia (highly expressing *Usp18*; Goldmann et al., 2015), and cluster 2 as DAM. (H) Feature plot of the custom senescence signature (*Cdkn2a*, *Cdkn1a*, *Cdkn2d*, *Casp8*, *Il1b*, *Glb1*, *Serpine1*) identifying the DAM cluster 2 as enriched in senescent genes. (I) Bar plots of the percentage of cells per genotype (left) or per cluster (right), with a low, medium, or high enrichment in the custom senescence signature (from all cells with senescence scores > 0). (J) Dot plot representing the expression of the individual genes of the custom senescence signature in the 4 clusters identified in (F). N = 3–4 (A–E).

We fluorescence-activated cell sorting (FACS) sorted the subpopulations of CD11C^+^ and CD11C^−^ microglia from 10-month-old APP/PS1 mice (as in Figure S2) and analyzed their transcriptomic profile by bulk RNA sequencing RNA-seq with the Smart-seq2 method (Picelli et al., 2013). We found 164 differentially expressed genes (DEGs; p < 0.01) in the CD11C^+^ microglial population, when compared with the CD11C^−^ population, supporting the profound phenotypic change of microglia induced in the APP/PS1 model (Figure 3A). Our data showed correlation (R = 0.54) with the top 100 genes, with highest and lowest fold change of DAM compared to homeostatic microglia (Keren-Shaul et al., 2017) (Figure 3B), confirming that the CD11C^+^ cells isolated and analyzed here are indeed DAM.

To further understand the profile of DAM, we correlated our RNA-seq data from DAM and homeostatic microglia with published datasets defining the senescence-associated gene expression signatures of different cell types (Hernandez-Segura et al., 2017) (Figures 3C and 3D). The key genes defining the core senescence signature shared by four independent cell types (melanocytes, keratinocytes, astrocytes, and fibroblasts) showed positive correlation with the signature observed in DAM (Figures 3C and 3D). This correlation was most pronounced when DAM were compared to WT microglia (R = 0.24) and still evident when DAM were compared to homeostatic microglia from APP/PS1 mice (0.20) (Figure 3D). We then performed gene set enrichment analysis (GSEA) (Mootha et al., 2003; Subramanian et al., 2005) using seven published signatures of genes upregulated or downregulated in senescent cells (Hernandez-Segura et al., 2017; Fridman and Tainsky, 2008; Casella et al., 2019; Kamminga et al., 2006), as well as a custom list of genes accepted to increase in senescent cells (*Cdkn2a*, *Cdkn1a*, *Cdkn2d*, *Casp8*, *Il1β*, *Cxcl8*, *Glb1*, *Serpine1*) (Figure 3E). When comparing DAM (CD11C^+^) versus homeostatic microglia (CD11C^−^) from APP/PS1 mice, we observed positive enrichment (positive normalized enrichment score [NES]) of those gene sets classically upregulated in senescent cells and negative enrichment (negative NES) of those repressed in senescent cells (Figure 3E). This analysis validated the correlation analysis (Figures 3C and 3D) and also confirmed the association of DAM with a senescent transcriptional profile.

To further explore the association of DAM with a senescent phenotype, we analyzed a single-cell dataset from 16-month-old APP/PS1 and WT mice, initially described by Van Hove et al. (2019). We identified a total of 4 microglial clusters, and annotated cluster 1 as homeostatic microglia in C57BL/6 mice, cluster 0 as homeostatic microglia in APP/PS1 mice, cluster 3 as white matter microglia (due to high expression of *Usp18* (Goldmann et al., 2015), and cluster 2 as DAM (Figures 3F, 3G, and S5). Our analysis was concordant with the previously reported clustering by Van Hove et al. (2019), displaying an overlap of the DAM annotation (Figures 3G and S5C). We probed the dataset for enrichment of the custom senescence signature (see above; Figure 3E), identifying an association of the DAM clusters with senescence genes (Figure 3H). We observed a significant number of cells displaying a senescence signature associated with APP/PS1 mice, in particular with clusters 0 and 2, both associated with this genotype (Figure 3I). Analysis of the enrichment of the individual genes from the custom senescent signature highlighted *Cdkn1a, Glb1*, and *Serpine1* as highly enriched in the DAM clusters (Figure 3J). These results indicate that DAM display a gene expression signature that is characteristic of senescent cells.

### Human microglia display markers of senescence in AD

**Figure 4:**
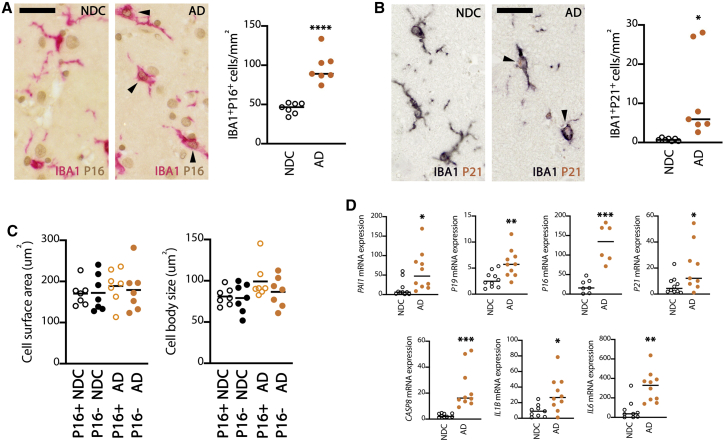
Microglial senescence in human AD (A and B) Analysis of the expression of cell-cycle inhibitors associated with senescence P16 (A) and P21 (B) in microglia (IBA1^+^) in the gray matter of the temporal cortex of human AD cases and non-demented controls (NDCs). Density of IBA1^+^P16^+^ or IBA1^+^P21^+^ cells represented as means ± SEMs. Representative immunopositive cells identified with an arrowhead. (C) Morphological analysis of P16^+^ microglia (Iba1^+^) in the gray matter of the temporal cortex of human AD cases and NDCs. (D) mRNA expression of selected markers of senescence (*PAI1*, *P19*, *P16*, *P21*, *CASP8*) or senescence-associated secretory patters (SASP; *IL-1β, IL-6*), in the temporal cortex of human AD cases and NDCs. Data represented as means ± SEMs and indicated as relative expression to the normalization factor (geometric mean of 4 housekeeping genes; HPRT and GUSB) using the 2-ΔΔCT method. N = 10. Scale bars in (A) and (B), 40 μm. N = 6–7 (A–C). Statistical differences: ^∗^p < 0.05, ^∗∗^p < 0.01, ^∗∗∗^p < 0.001, and ^∗∗∗∗^p < 0.0001 versus NDC. Data were analyzed with an unpaired t test (A and B) or with a 2-tailed Fisher t test with correction for multiple comparisons (C).

We next studied microglial senescence in two independent cohorts of human post-mortem samples from patients with AD and age-matched non-demented controls (NDCs), by staining for associated cell-cycle inhibitors (Figure 4). In the gray matter of the temporal cortex of AD cases we found a significant increase in the density of P16^+^ microglia (IBA1^+^), localizing in the nuclear compartment (Figure 4A). Detailed analysis following co-staining with ThioS indicated that 40.46% ± 5.23% of P16^+^IBA1^+^ cells were associated with Aβ plaques. Similarly, we found a significant increase in the density of P21^+^IBA1^+^ cells in AD when compared to NDCs (Figure 4B). Detailed morphological analysis did not detect any significant change in microglial surface area or body size associated with P16 expression, although a trend toward enlarged body size was noted in P16^+^ microglia in AD cases (Figure 4C). In an independent cohort, we found a significant increase in the mRNA expression of markers associated with senescence, including *PAI1*, *P19*, *P16*, *P21*, and CASPASE-8 *(CASP8)*, as well a significantly increased expression of *IL-1β* and *IL-6*. Although these genes, in isolation, are not unequivocal markers of senescence, the upregulation of this panel is characteristic of a senescence-associated secretory pattern (SASP) (Figure 4D) and highly supportive of a senescent phenotype.

Our data indicate that senescent microglia can be found in the APP/PS1 model of AD-like pathology and in human AD. DAM are senescent cells, displaying several characteristic features, including SA-βgal activity, telomere shortening, and a senescence-associated transcriptional profile.

### Inhibition of early microglial proliferation prevents the onset of microglial senescence and ameliorates amyloid-related pathology

**Figure 5:**
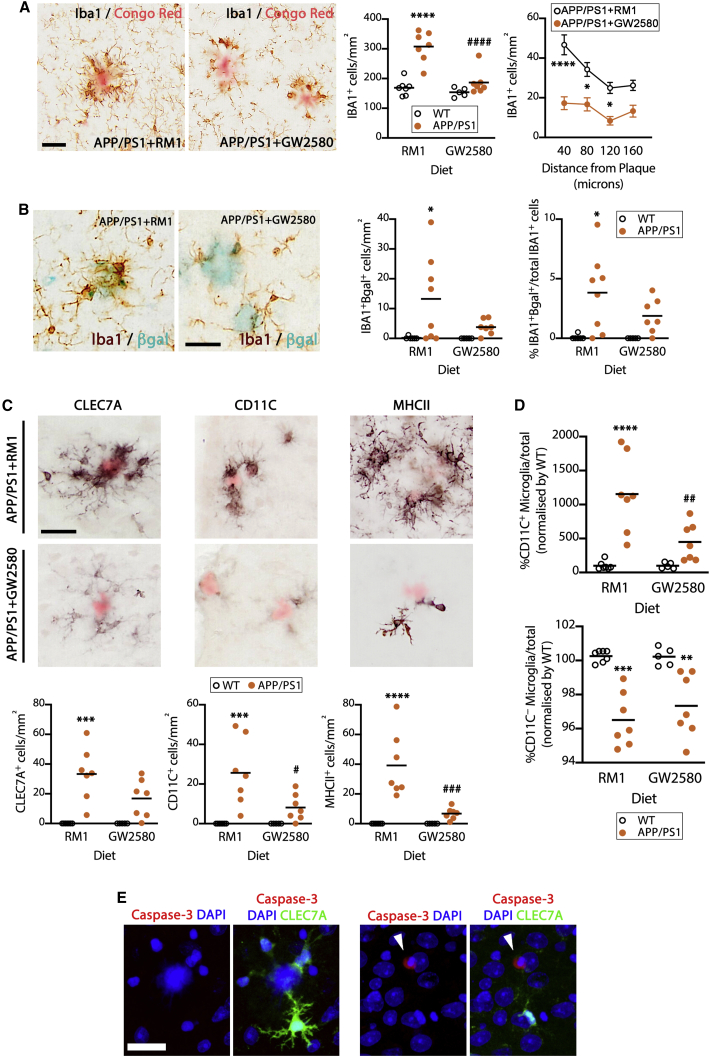
Prevention of microglial proliferation impairs the development of DAM (A) Microglial (IBA1^+^) density and plaque association in APP/PS1 mice and WT littermates after treatment with a diet containing GW2580 (CSF1R inhibitor) or a control diet (RM1) for 4 months from the pre-plaque stage (3.5 months of age), analyzed by IHC (brown). Aβ plaques labeled with Congo Red. (B) Density and frequency (percentage of total microglial population) of senescent microglia (βgal^+^, blue; IBA1^+^, brown) in APP/PS1 mice and age-matched controls, after treatment as in (A), analyzed by IHC. (C) Density of DAM, identified as CLEC7A^+^, CD11C^+^, or MHCII^+^ cells, in APP/PS1 and WT littermate controls, after treatment as in (A), analyzed by IHC (black). Aβ plaques labeled with Congo Red. (D) Flow cytometry analysis of the frequency of DAM (CD11B^+^CD45^low^CD11C^+^) and homeostatic microglia (CD11B^+^CD45^low^CD11C^−^) in APP/PS1 and WT controls, after treatment as in (A). (E) Confocal imaging of the co-localization of cleaved caspase-3 (red) and CLECL7A (green) in APP/PS1 mice treated with GW2580 as in (A). Nuclei stained with DAPI, shown in blue. Representative example of a caspase-3^+^CLEC7A^−^ cell identified with an arrowhead (right). IHC data collected from average of parietal, auditory, and entorhinal cortex. Scale bars in (A)–(C) and (E), 40 μm. Flow cytometry performed in samples from the cerebral cortex. Data shown in (A)–(D) represented as means ± SEMs. N = 5–7. Statistical differences: ^∗^p < 0.05, ^∗∗^p < 0.01, ^∗∗∗^p < 0.001, and ^∗∗∗∗^p < 0.0001 versus age-matched control; ^#^p < 0.05, ^##^p < 0.01, ^###^p < 0.001, and ^####^p < 0.0001 versus RM1 group. Data were analyzed with a 2-way ANOVA and post hoc Tukey tests.

**Figure 6:**
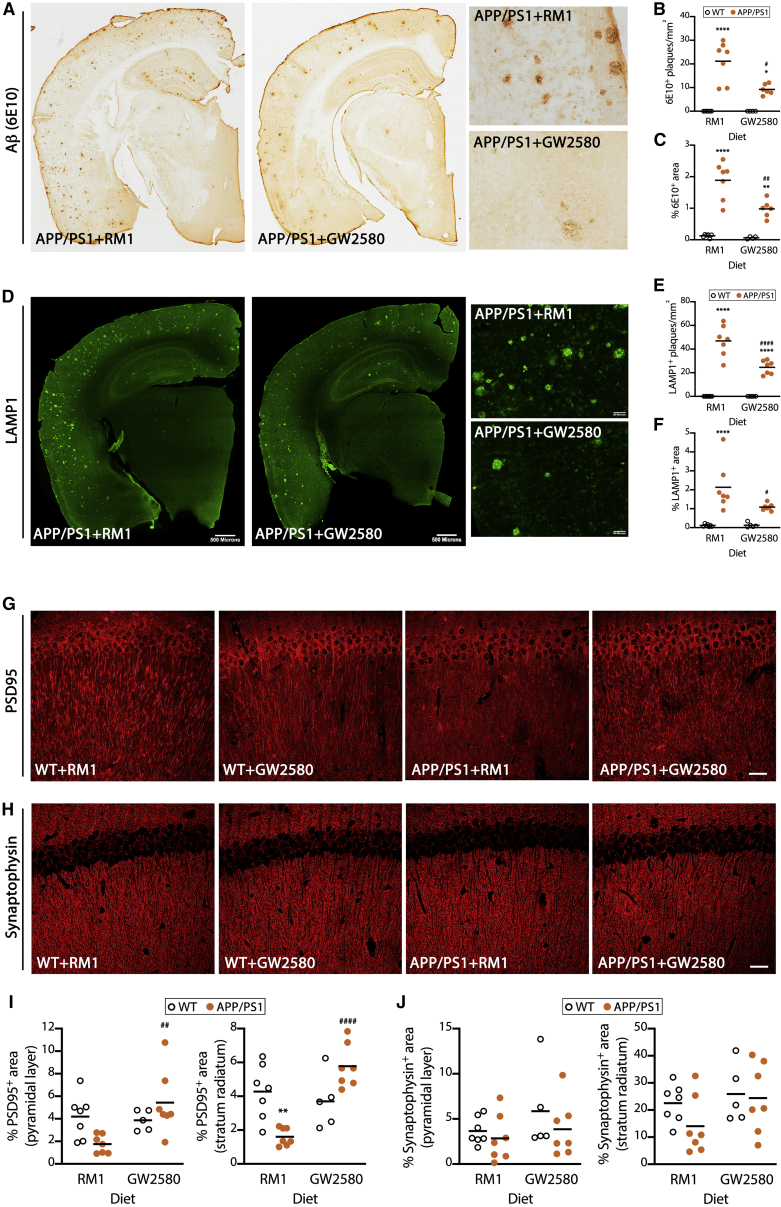
Prevention of microglial senescence ameliorates amyloid-related pathology (A–C) Analysis of the amyloid pathology, quantified as density (B) and area covered (C) of Aβ plaques (6E10^+^, brown) in APP/PS1 mice and age-matched controls, after treatment with a diet containing GW2580 (CSF1R inhibitor) or a control diet (RM1) for 4 months from the pre-plaque stage (3.5 months of age), analyzed by IHC. Representative overview and detailed images shown in (A). (D–F) Analysis of the axonal dystrophy pathology, quantified as density (E) and area covered (F) of LAMP1^+^ plaques (green) in APP/PS1 mice and age-matched controls, after treatment as in (A)–(C), analyzed by IHC. Representative overview and detail images shown in (D). (G–J) Analysis of the synaptic pathology, quantified as optical density of PSD95 (post-synaptic marker; G and I) or synaptophysin (post-synaptic marker; H and J) in APP/PS1 mice and age-matched controls, after treatment as in (A)–(C), analyzed by IHC. IHC data collected from an average of parietal, auditory, and entorhinal cortex (Aβ and LAMP1) or hippocampus (pyramidal layer and stratum radiatum). Scale bars in (A), (D, overview), 500 μm, shown in (D); (A) and (D, detail), 50 μm, shown in (D); (G) and (H), 50 μm. Data shown in (B)—(F) represented as means ± SEMs. N = 5–7. Statistical differences: ^∗^p < 0.05, ^∗∗^p < 0.01, and ^∗∗∗∗^p < 0.0001 versus age-matched controls; ^#^p < 0.05, ^##^p < 0.01, and ^####^p < 0.0001 versus RM1 group. Data were analyzed with a 2-way ANOVA and post hoc Tukey tests.

Our original hypothesis was that elevated microglial proliferation in AD contributes to the pathology. Therefore, to address whether these detrimental effects could be ameliorated by restricting microglial proliferation from the outset, we performed experiments with a non-depleting dose of a CSF1R inhibitor (GW2580) intervening to prevent the expansion of microglia observed immediately after plaque onset (Figure 5A). Under these conditions, microglial density remains at WT levels, with the most profound changes observed in the plaque-associated subpopulation (Figure 5A). Inhibition of microglia proliferation prevented the onset of microglial senescence, as seen in a decreased density and frequency of IBA1^+^βgal^+^ cells (Figure 5B). GW2580 also prevented the increase in DAM, identified as CLEC7A^+^, CD11C^+^, or MHCII^+^ cells (Figure 5C). The prevention of microglial proliferation caused a selective reduction in the DAM (CD11C^+^), not seen in the population of homeostatic microglia (CD11C^−^) (Figure 5D). Analysis of the expression of the apoptosis marker cleaved caspase-3 did not demonstrate any pro-apoptotic effect of the GW2580 treatment in CLEC7A^+^ cells (Figure 5E, left), while caspase-3^+^CLEC7A^−^ cells could be found in APP/PS1 mice (Figure 5E, right). We validated this effect of GW2580 on the DAM population by using the ME7 model of prion disease (Figure S6A), as an alternative model of chronic neurodegeneration with well-characterized microgliosis (Gómez-Nicola et al., 2013) but lacking Aβ pathology. We found a significant effect of GW2580 on the density of DAM (CLEC7A^+^ cells; Figure S6B), which in this model represents 85% of the microglial population. This suggests that the effect of GW2580 on the DAM population is direct, instead of secondary to the effects on Aβ levels. Notably, in the APP/PS1 model, the selective prevention of the conversion of microglia to DAM, via proliferation, caused a significant prevention of the amyloid pathology, evidenced by a reduction in the density of Aβ plaques and total Aβ load (Figures 6A–6C). This was accompanied by a significant reduction in the axonal pathology associated with the Aβ plaques, in the form of reduced density and load of LAMP1^+^ dystrophic neurites (Figures 6D–6F). To further understand the impact of the prevention of microglial senescence in APP/PS1 mice, we analyzed their synaptic pathology by quantifying the density of post- and pre-synaptic markers (Figures 6G–6J). The inhibition of microglial senescence and reduced conversion to DAM caused a significant preservation of the post-synaptic (PSD95^+^) compartment, otherwise significantly degenerated in APP/PS1 mice treated with a control diet (Figures 6G and 6I). We did not observe any significant degeneration of the pre-synaptic compartment (synaptophysin^+^) in APP/PS1 mice (Figures 6H and 6J), probably due to the relatively early stage of the progression. Overall, our data indicate that excessive microglial proliferation drives the phenotypic specification of DAM, via the onset of replicative senescence, which accelerates amyloid-related pathology.

## Discussion

Over the last decade, a substantial body of evidence has placed microglia, alongside neuroinflammation, center stage in the pathophysiology of AD, with relevant therapeutics moving rapidly through the preclinical pipeline (Martin-Estebane and Gomez-Nicola, 2020). The field now agrees that microglia likely undergo phenotypic changes during the development and progression of the underlying pathology, but we lack a clear understanding of the mechanisms stimulating and driving these changes. One model suggests that the detection of early, pre-clinical pathology triggers a microglial proliferative response, and this places microglia on an independent trajectory to accelerate and execute disease progression (Simon et al., 2019). Here, we tested the hypothesis that this early proliferation unleashes subsequent replicative senescence, determining the specification of disease-associated microglial phenotypes that drive the damaging inflammatory milieu characteristic of AD. Our results support this hypothesis, and indicate that DAM (Keren-Shaul et al., 2017; Krasemann et al., 2017) are generated as a consequence of early microglial proliferation and are phenotypically characterized by a senescence-associated profile. We demonstrate that (1) a subpopulation of DAM displays signs of replicative senescence in a model of AD-like pathology, including increased SA-βgal activity, a senescence-associated transcriptional signature, and telomere shortening; (2) senescent microglia are present in human AD; and (3) the prevention of early microglial proliferation hinders the development of senescence and DAM generation, with a direct impact on the prevention of Aβ pathology and synaptopathy. These results directly affect our mechanistic understanding of the phenotypic specification of microglia in AD, and offer routes for selective targeting of these unique senescent subpopulations in a clinical setting.

The study of microglia in AD has for many years provided evidence for the onset of senescence-associated changes in the brain. However, the unequivocal identification of senescence is challenging and elusive, as several mechanisms coalesce to present with a similar senescent phenotype (Carnero, 2013). The study of cultured microglia helped identify decreased telomerase activity associated with proliferation (Flanary and Streit, 2004), related to the dystrophic morphological microglial phenotype that correlates with intraneuronal neurofibrillary degeneration (Streit et al., 2020). Similar morphological alterations in microglia are observed in Parkinson’s disease (Shaerzadeh et al., 2020), highlighting the similarities in the response of these cells in chronic neurodegeneration. However, these morphological alterations, broadly categorized as dystrophy, could be attributed to several mechanisms and are not exclusive to senescence. These descriptive features were echoed by recent functional studies in models of AD-like pathology. Both astrocytes and microglia show signs of senescence in the MAPT^P301S^PS19 model of tau pathology, with pro-apoptotic agents such as most senolytics, having a positive impact on the overall pathology (Bussian et al., 2018). The detrimental role of astrocytic senescence has been validated *in vitro* recently (Limbad et al., 2020), supporting the significance of this response. Similarly, senolytic agents have a positive impact in the APP/PS1 model, primarily via affecting senescent oligodendrocyte precursor cells (OPCs) (Zhang et al., 2019). Our study provides a comprehensive assessment of the multiple aspects involved in the onset of senescence in microglia and identify excessive replication as the main driver for inducing senescence in these cells. The association between microglial proliferation and the induction of replicative senescence has been explored. For example, we reported that the microglial population in the dentate gyrus has a higher turnover rate when compared to other regions, leading to deficient replication in aging (Askew et al., 2017). Microglia in the dentate gyrus also show telomere shortening, as highlighted using a mouse model of telomere dysfunction (TERC KO) (Khan et al., 2015), therefore supporting the hypothesis that an elevated number of cycling events could trigger replicative exhaustion. A subpopulation of microglia (called dark microglia) display signs of pronounced oxidative stress and chromatin remodeling in APP/PS1 mice (Bisht et al., 2016). These dysfunctional microglia, reminiscent of DAM, are associated with the increased production of inflammatory cytokines and reactive oxygen species (ROS), alongside an impaired ability to regulate increased oxidative stress in the aging brain, and impaired phagocytosis, which ultimately accelerates neurodegeneration (Rawji et al., 2016). This is supported by studies in G3 mTerc^−/−^ mice, which display microglia with shortened telomeres and morphological dystrophy, leading to an enhanced pro-inflammatory response of microglia to lipopolysaccharides (LPS) (Raj et al., 2015). Our data support that DAM are senescent microglia, with an altered phenotypic and transcriptional profile directly affecting disease progression. In future studies, it would be interesting to define the type of senescence experienced by astrocytes and OPCs, as well as the mechanisms triggering it, to understand whether excess replication is exclusive to microglia or is indeed the main driver for all cell types exhibiting cellular senescence.

In regard to new therapeutic options based upon our findings, we propose that more refined targeting approaches will be required beyond the use of broad-spectrum senolytics, since these are mostly pro-apoptotic agents with associated risks when used in the context of an already degenerating brain, full of endangered neurons and glia. For example, based on our data indicating that DAM are senescent microglia, the initial identification of the mechanistic dependence of DAM on TREM2 and APOE function (Keren-Shaul et al., 2017; Krasemann et al., 2017) could now be interpreted as an increased vulnerability of these cells to the deprivation of pro-survival factors, since this pathway is, together with CSF1R, key in controlling microglial survival (Wang et al., 2015). Accordingly, two recent studies using microglia depletion strategies via high doses of CSF1R inhibitors in the 5xFAD AD model showed that pre-plaque microglial depletion is sufficient to prevent the onset of plaque pathology (Sosna et al., 2018; Spangenberg et al., 2019) via unknown mechanisms. Here, we show that reducing the dose of CSF1R inhibitors to elicit microglial growth inhibition but not depletion is sufficient to induce this beneficial effect, linked to its ability to prevent microglial senescence and DAM formation. At first glance, these results are in contradiction to what we and others reported previously, when we observed that late-stage inhibition of CSF1R had no impact on plaque pathology, despite driving a beneficial impact on synaptic preservation and overall pathology in models of amyloidosis (Olmos-Alonso et al., 2016; Spangenberg et al., 2016; Dagher et al., 2015) or tau pathology (Mancuso et al., 2019). However, here, we implemented early intervention with the CSF1R inhibitor. This suggests a biphasic interaction of microglia with Aβ, whereby microglia, and in particular DAM, participate in the early events of plaque formation, to later engage in an Aβ-independent trajectory that affects synaptic and neuronal damage. More broadly, our study was limited to the initial amyloid-related changes, as we did not study the impact of these mechanisms on cognitive decline or the activity-related changes commonly observed in models of AD-like pathology. We acknowledge this as a limitation, which will need to be addressed in future, more chronic interventions. A better understanding of the influence of microglia in early plaque seeding and spreading is important, then, to understanding the earliest events leading to pathology. An aspect of particular interest would be to reconcile the previously reported increased phagocytic activity of DAM (Keren-Shaul et al., 2017) with the pro-amyloid effect observed here, unveiled after impeding DAM formation. One possibility would be the existence of different subpopulations of DAMs, with different roles and phagocytic ability, which may (or may not) coexist in time. Our present findings already indicate that the DAM population is heterogeneous, as suggested by the relative presence of senescence markers in subgroups of DAMs. In summary, it is now critical to explore potential ways to specifically target the disease-associated, senescent microglia in AD, as a route toward an efficacious treatment to prevent subsequent pathology.

## STAR★Methods

### Key resources table

**Table:** 

REAGENT or RESOURCE	SOURCE	IDENTIFIER
**Antibodies**		

Rabbit anti-Iba1	In-house (Covalab)	from Imai et al.,1996
Mouse anti-CDKN2A/p16INK4a	Abcam	Cat# ab54210; RRID:AB_881819
Mouse anti-p21	Santa Cruz Biotechnology	Cat# sc-6246; RRID:AB_628073
Mouse anti-amyloid-β	Covance	6E10; Cat# SIG-39330-200; RRID:AB_662804
Rat anti-MHC class II (I-A/I-E)	ThermoFisher	Cat# 14-5321-85; RRID:AB_467562
Rat anti-Dectin1	InvivoGen	Cat# mabg-mdect; RRID:AB_2753143
Hamster anti-CD11c	Bio-Rad	Cat# MCA1369GA; RRID:AB_324695
Rat anti-LAMP1	DSHB	Cat# 1d4b; RRID:AB_2134500
Rabbit anti-PSD95	Frontiers Institute	Cat# PSD95-Rb-Af628; RRID:AB_2571540
Rabbit anti-synaptophysin	Abcam	Cat# ab32127; RRID:AB_2286949
(BV) 421 anti- CD11b	eBioscience	clone M1/70
PE anti-mouse CD11c	Biolegend	clone N418
APC-anti mouse Clec7a	Biolegend	clone R1-8g7
Alexa488 CD11b	eBioscience	clone M1/70
BV785 CD45	Biolegend	clone 30F-11
BV421 CD11c	Biolegend	clone N418

**Biological samples**		

Human post-mortem brain sections	National CJD Surveillance Unit Brain Bank (Edinburgh, UK)	N/A
Human post-mortem fresh frozen brain samples	South West Dementia Brain Bank, University of Bristol (UK)	N/A

**Chemicals, peptides, and recombinant proteins**		

GW2580	LC Laboratories	G-5903
Thioflavin S	Merck	T1892
Bissulfoscuccinimidyl suberate (BS_3_)	Thermo Fisher Scientific	21580
Tel Cy5 probe (AATCCC)n probe	Panagene Inc.	F1003

**Deposited data**		

Mouse RNaseq data from isolated microglia	This paper	Synapse (Synapse ID: syn25567929)
Mouse single-cell RNaseq data	Van Hove et al., 2019	https://www.brainimmuneatlas.org/

**Experimental models: cell lines**		

T1301 cells	Culture Collections, Public health England, UK	N/A
Jurkat T cells	ATCC	Cat# TIB-152; RRID:CVCL_0367

**Experimental models: organisms/strains**		

APPswe/PSEN1dE9 mice (APP/PS1) mice	Jackson Laboratory	34832-JAX
c-fms EGFP “Macgreen” mice	Laboratory of David Hume	Sasmono et al., 2003
C57BL/6J mice	Harlan	

**Oligonucleotides**		

Primers for RT-PCR, see RT-PCR methods section	This paper	N/A
Primer for APP genotyping (forward: GAATTCCGACATGA CTCAGG)	This paper	N/A
Primer for APP genotyping (reverse: GTTCTGCTGCATCTTGGACA)	This paper	N/A

**Software and algorithms**		

Prism 8	GraphPad	https://www.graphpad.com/scientific-software/prism/
ImageJ	ImageJ	https://imagej.nih.gov/ij/
Seurat (v3.2.2)	Liao et al., 2014; Love et al., 2014.	https://satijalab.org/seurat/
GSEA	Mootha et al., 2003; Subramanian et al., 2005.	https://www.gsea-msigdb.org/gsea/index.jsp
DIVA™ 8	BD Biosciences	https://www.bdbiosciences.com/en-us/instruments/research-instruments/research-software/flow-cytometry-acquisition/facsdiva-software
FlowJo X 10.8	Flowjo	https://www.flowjo.com
SDS v.2.0.6	ThermoFisher	https://www.thermofisher.com/us/en/home/technical-resources/software-downloads/applied-biosystems-7500-real-time-pcr-system.html

### Resource availability

#### Lead contact

Further information and requests for resources and reagents should be directed to and will be fulfilled by the lead contact, Diego Gomez-Nicola (d.gomez-nicola@soton.ac.uk).

#### Materials availability

This study did not generate new unique reagents.

#### Data and code availability

Mouse RNA-seq data that support the findings of this study have been deposited in Synapse (Synapse: syn25567929).

### Experimental model and subject details

#### Experimental mice

APPswe/PSEN1dE9 mice (APP/PS1) on a C57BL/6 background were originally obtained from the Jackson Laboratory (Jankowsky et al., 2004). APP/PS1 heterozygous males were bred at our local facilities with wild-type (WT) female C57BL/6J (Harlan) or c-fms EGFP “Macgreen” female mice (Sasmono et al., 2003), allowing the c-fms EGFP transgene to be expressed in heterozygotes. Offspring were ear punched and genotyped using PCR with primers specific for the APP-sequence (forward: GAATTCCGACATGA CTCAGG, reverse: GTTCTGCTGCATCTTGGACA). Mice not expressing the transgene were used as WT littermate controls. Mice were housed in groups of 4 to 10, under a 12-h light/12h dark cycle at 21°C, with food and water *ad libitum*.

To characterize DAMs and senescence, APP/PS1, APP/PS1/Macgreen mice and their WT littermate controls were sacrificed at 4, 6, 10 and 12-13 months of age (n = *5 mice/group*). To determine the effects of the drug GW2580 (LC Laboratories) on pre-plaque pathology, APP/PS1, APP/PS1 Macgreen mice and their WT littermate controls were fed from 3.5 months of age, either normal diet (RM1) or diet with GW2580 (SAFE Nutrition Ltd. (1500 ppm) (n = *5-7 mice/group*). Treatment lasted 4 months after which animals were sacrificed by terminal perfusion-fixation.

To induce prion disease 6 weeks old C57BL/6J mice were anesthetized with a ketamine/xylazine mixture (85 and 13 mg/kg), and 1 μL of ME7-derived (ME7 group) brain homogenate (10% w/v) was injected stereotaxically and bilaterally at the dorsal hippocampus, coordinates from bregma: anteroposterior, −2.0 mm; lateral, ± 1.7 mm; depth, −1.6 mm. Mice injected with NBH (normal brain homogenate) were used as controls.

Experimental groups were designed ensuring we had a balanced number of male and female mice, and no sex-specific effect was observed when performing sub-analysis of our data. Mouse weight was monitored throughout the experiment. All procedures were performed in accordance with Home Office regulations.

#### Post-mortem human brain samples

For immunohistochemical (IHC) analysis, human brain autopsy tissue samples (temporal cortex, paraffin-embedded, formalin- fixed, 96% formic acid-treated, 6-mm sections) from the National CJD Surveillance Unit Brain Bank (Edinburgh, UK) were obtained from cases of AD and age-matched controls (Table S1), in whom consent for use of autopsy tissues for research had been obtained. All cases fulfilled the criteria for the pathological diagnosis of AD (Table S1). Ethical permission for research on autopsy materials stored in the National CJD Surveillance Unit were obtained from Lothian Region Ethics Committee.

For mRNA analysis, human brain autopsy tissue samples (temporal cortex, fresh-frozen tissue) were obtained from the Human Tissue Authority licensed South West Dementia Brain Bank, University of Bristol (UK). Samples were selected from AD cases and age-matched controls (Olmos-Alonso et al., 2016). Ethical permission for research on autopsy materials stored in the South West Dementia Brain Bank was obtained from Local Ethics Committee.

#### Cell lines

T1301 cells (Culture Collections, Public health England, UK) and Jurkat T cells (ATCC cat#TIB-152), two lines of human T cell leukemia were used as an internal reference control for the measurement of telomere length by Flow-FISH. Cells were cultured according to the manufacture’s conditions, in RPMI 1640 (GIBCO, Thermo Fisher Scientific) supplemented with 10% fetal bovine serum (GIBCO, Thermo Fisher Scientific), 2 mM L-glutamine, 100U/ml penicillin and 100 μg/ml streptomycin. Importantly, subculture of T1301 cells did not exceed 4 passages.

### Method details

#### Analysis of gene expression by RT-PCR

Frozen samples from AD cases or age-matched controls were processed for RNA extraction and qPCR analysis. RNA was extracted using the RNAqueous-Micro Kit (Life Technologies), quantified using Nanodrop (Thermo Scientific), to be retro-transcribed using the iScript cDNA Synthesis Kit (Bio-Rad), following manufacturer’s instructions, after checking its integrity by electrophoresis on a 1.8% agarose gel. Low quality or purity RNA samples were excluded from consequent experimentation. cDNA libraries were analyzed by qPCR using the iTaq Universal SYBR Green supermix (Bio-Rad) and the following custom designed gene-specific primers (Sigma-Aldrich): *l1b* (NM_008361.3; FW, 5′- GAAATGCCACCTTTTGACAGTG-3′, RV 5′-TGGATGCTCTCATCAGGACAG-3′), *Il6* (NM_031168.1; FW, 5′-TAGTCCTTCCTACCCCAATTTCC-3′, RV, 5′-TTGGTCCTTAGCCACTCCTTC-3′), *Casp8* (NM_001080126.1; FW, 5′-TGCCTCCTCCTATGTCCTGT-3′, RV, 5′-GAGGTAGAAGAGCTGTAACCTTATC-3′), *Pai1* (NM_008871; FW, AAGTCTTTCCGACCAAGAGCA-3′, RV, 5′-GGTTGTGCCGAACCACAAAG-3′), *p19* (NM_009878; FW, 5′-GCTCTGAGGCCGGCAAAT-3′, RV, 5′- TCATGACCTGCAAGGCCGTC-3′), *Gapdh* (NM_008084.2; FW, 5′-TGAACGGGAAGCTCACTGG-3′, RV, 5′-TCCACCACCCTGTTGCTGTA-3′), *Hprt* (NM_013556.2; FW, 5′-CAGTCCCAGCGTCGTGATTA-3′, RV, 5′-TGGCCTCCCATCTCCTTCAT- 3′), and *Ppia* (NM_008907.2; FW, 5′-AGGGTGGTGACTTTACACGC-3′, RV, 5′-CTTGCCAGCCATTCAG- 3′). Quality of the PCR reaction end product was evaluated by electrophoresis in a 1.5% agarose gel. Raw CT data were obtained from the SDS v.2.0.6 software and normalized to the normalization factor (geometric mean of three housekeeping genes; GAPDH, HPRT, and PPIA) using the 2-ΔΔCT method.

#### Immunohistochemistry (IHC)

Coronal hippocampal sections were cut on a vibratome from 4% paraformaldehyde-fixed, frozen or fresh brains. Mice perfusion, tissue processing and immunohistochemical analysis was performed as previously described (Olmos-Alonso et al., 2016; Gómez-Nicola et al., 2013). Sections were incubated with primary antibodies: rabbit anti-Iba1 (Covalab; 1:1000; (Imai et al., 1996)), mouse anti-CDKN2A/p16INK4a antibody [2D9A12] (abcam, ab54210; 1:1000), mouse anti-p21 (Santa Cruz Biotechnology, sc-6246 (F-5); 1:500), mouse anti-amyloid-β (6E10; Covance; 1:500) (pre-treatment with 80% formic acid for 10 minutes), rat anti-MHC Class II (I-A/I-E) (ThermoFisher, 14-5321-85;1:500), rat anti-Dectin1 (InvivoGen, mabg-mdect; 1:200), hamster anti-CD11c, N418 (Bio-Rad, MCA1369GA; 1:500), rat anti-LAMP1 (DSHB, 1D4B; 1:100), rabbit anti-PSD95 (Frontiers Institute, RbAf628, 1:200), rabbit anti-Synaptophysin (Abcam, ab32127, 1:1000). Following incubation with primary antibodies, sections were washed and incubated with the corresponding biotinylated secondary antibody (Vector Labs) or ImmPress-AP kit (Vector Labs) for bright field IHC. For fluorescent IHC, sections were incubated with Alexa Fluor 488 or 568 conjugated secondary antibodies or streptavidin 647 (Molecular Probes). Brightfield IHC was developed with 3,3′-diaminobenzidine (DAB) precipitation (brown) alone or combined with 0.05% nickel ammonium sulfate for contrast (black), followed by BCIP/NBT (Vector) alkaline phospatase (blue/purple) reaction and 1% congo red to visualize amyloid plaques, then dehydrated and mounted with Depex. DAPI was used as counterstain in fluorescent IHC, then mounted with Mowiol/DABCO. Human sections followed the same protocols with the addition of dewaxing and antigen retrieval in citrate buffer for 25 minutes (Olmos-Alonso et al., 2016). For detection of Aβ plaques in human tissue, sections were incubated with 1% solution of Thioflavin S (Merck) in 80% ethanol for 8 minutes, followed by rinsing twice in 80% ethanol, before incubation in 0.2M PBS for 30 minutes.

#### Senescence associated β-galactosidase activity

Methods to analyze β-gal utilize the enzymatic activity releasing an insoluble blue product when the endogenous enzyme hydrolyses X-gal in solution, as method used to detect cellular senescence (Dimri et al., 1995; Lee et al., 2006). Sections were first washed in X-gal buffer (50ml 5mM EGTA pH8, 0.4g Magnesium chloride (MgCl_2_·6H_2_O) 0.4ml 0.04% NP40, 0.1g Deoxychoic sodium) made in 1L PBS 1M, and adjusted to pH6 before use. Sections were stained with pH6 X-gal staining solution (25ml X-gal buffer, 0.045 g potassium ferricyanide (K_3_Fe), 0.06 g potassium ferrocyanide trihydrate (K_4_Fe) and 250ul x-gal enzyme (1:1000; stock at 50mg/ml; 0.5mg/ml in solution)) at 37°C on a shaker for 6-9 hours. To stop the reaction, sections were washed in X-gal buffer followed by post fixation with 4% PFA for 10mins, followed by IHC for IBA1.

#### Microglial fluorescence-activated cell sorting (FACS)

APP/PS1/Macgreen and WT control mice were terminally anesthetized with pentobarbital, followed by transcardial perfussion with ice-cold heparinized phosphate buffered saline (GIBCO, Thermo Fisher Scientific, pH7.4) without Ca^2+^ or Mg^2+^. The brain was removed from the skull and brain samples containing cortex and hippocampus were collected. After mechanical dissociation the tissue was subjected to enzymatic dissociation with collagenase (300 units/ml, Worthington) and DNase I (50 μg /ml, Sigma) at 37 ^◦^C for 1 hour, to later pass the cell suspension through a 70 μm cell strainer. The suspension was purified by centrifugation in density gradient of 37% percoll (GE Health) at 500 g for 30 min at 18°C, discarding the myelin-enriched supernatant. The cell pellet, enriched in microglia, was re-suspended in FACS buffer containing PBS, 1% fetal calf serum and 2mM EDTA. The purified cell suspension was labeled with brilliant violet (BV) 421 anti- CD11b (clone M1/70, 1:400; eBioscience), PE anti-mouse CD11c (clone N418, 1:100; Biolegend) and/or APC-anti mouse Clec7a (clone R1-8g7, 1:100; Biolegend), while 7-aminoactinomycin D (7-AAD) was added as a cell viability marker. Negative control samples (not stained) were used to set the fluorescence thresholds for each marker. Cells were sorted using a BD FACS Aria Flow cytometer and collected into a nuclease free collection tube (Thermo #3453), followed by storage at −80°C until processing. For FACS analysis, 100,000 events were recorded, later analyzed using Flowjo software version 10.8.

#### Measurement of telomere length by flow cytometry *in situ* hybridization (FLOW-FISH)

Microglia purification and isolation were performed as described above, with cells being labeled with Alexa488 CD11b (clone M1/70, eBioscience), BV785 CD45 (clone 30F-11, Biolegend) and BV421 CD11c (clone N418, Biolegend) on ice for 45 minutes. To improve the stability of antigen-antibody-conjugate complexes, the cells were fixed in 200μm bissulfoscuccinimidyl suberate (BS_3_) crosslinking solution (Thermo Fisher Scientific) in PBS for 30 minutes on ice. Residual BS_3_ was further quenched with 50mM Tris-HCl in PBS at room temperature for 20 minutes, followed by wash with PBS to remove excess Tris-HCl from the samples. Brain cells were mixed with the same cell number of fixed T1301 cells, and the cell mixture was split into two equal aliquots. One aliquot of the mixed cells was re-suspended in hybridization buffer (70% deionized formamide, 14.25mM Tris-HCl pH 7.2, 1.4% BSA and 0.2M NaCl) containing 15.2 ng/ml Tel Cy5 probe (AATCCC)_n_ (Panagene Inc.), and another aliquot was incubated with hybridization buffer, without the probe, to correct for possible formamide-associated Cy5 auto-fluorescence. The samples were heated at 82°C for 10 minutes to allow telomeric double strand DNA denaturation, followed by rapid cool down in ice. The samples were placed in a chamber at 23°C and hybridized for two hours in the dark. Cells were pelleted by centrifugation at 1500 g and washed twice with PBS containing 0.14% BSA at 40°C. Cells were counterstained with 0.1 μg/ml of propidium iodide (PI, Thermo Fisher Scientific) in PBS containing 0.14% BSA, 10μg/ml RNase (Sigma Life Sciences) and 0.1% Tween-20. Unstained control samples and single staining samples of each dye were set up to perform fluorescence compensation. For gating the cell populations, T1301, Jurkat or brain cells were prepared separately, to identify the location of the population in a FSC/SSC plot. Then, singlets in G_0_/G_1_ cell cycle phase were selected based on PI fluorescence. Finally, the cell count for Cy-5 (APC channel) for every population of interest was collected to measure the median fluorescence intensity (MFI) of the PNA probe hybridized to telomeric DNA. To standardize fluorescence intensity units of the cytometer, the quantitative fluorescence calibration assay was performed by using MESF Quantum^Cy5^ to create a linear calibration curve that related to instrument channel values. The stained cell samples were then acquired using the same fluorescence settings (PMT, voltage and compensation). The experiments were performed on a LSR Fortessa™ flow cytometer equipped with 405, 488, 561, 635 nm laser detectors. A minimum of 20, 000 events were acquired for each sample using DIVA™ 8 software (BD, San Jose, CA) and data was analyzed by FlowJo X 10.8.

To correct for inter-assay variation in the MFI of each sample, the Cy5 APC MFI of the microglia and the T1301 cells were collected, calculating the relative telomere length (RTL) of microglia as follows:

$${\text{MicrogliaTL}}_{\text{MFI}}/{\text{T}1301\text{TL}}_{\text{MFI}}=\left({\text{MFI}}_{\text{Cy}5}\left({\text{Microglia}}_{\text{probe}}\right)-{\text{MFI}}_{\text{Cy}5}\left({\text{Microglia}}_{\text{negative}}\right)\right)/(\left({\text{MFI}}_{\text{Cy}5}\left({\text{T}1301}_{\text{probe}}\right)-{\text{MFI}}_{\text{Cy}5}\left({\text{T}1301}_{\text{negative}}\right)\right)$$

#### RNaseq of microglia isolated by FACS

cDNA synthesis from small pools of cells was performed at the Oxford Genomics Centre (Wellcome Trust Centre for Human Genetics) following the Smart-seq2 method (Picelli et al., 2013) and libraries prepared using Nextera XT (Illumina) with 0.25ng cDNA input and 12 PCR cycles, as previously described (Askew et al., 2017). All libraries were pooled and sequenced on one lane of a HiSeq4000 at 75bp paired end. Reads were aligned to Mus_musculus.GRCm38 genome using STAR aligner (Dobin et al., 2013) and genes counted with featurecounts (Liao et al., 2014) using Mus_musculus.GRCm38.95.gtf annotation. Gene counts from each of the libraries were combined, normalized and used to calculating deferentially expressed genes using Deseq2 (Love et al., 2014).

#### Gene set enrichment analysis (GSEA)

We applied GSEA (Mootha et al., 2003; Subramanian et al., 2005) to determine whether *a priori* defined sets of genes show statistically significant, concordant correlation with the gene expression changes observed in DAM and Homeostatic microglia. The input data for the GSEA were the following: (1) a complete table of gene reads from our RNaseq experiment (see above), (2) a mapping file for identifying probesets, and (3) signatures upregulated or downregulated in senescence cells (Hernandez-Segura et al., 2017; Fridman and Tainsky, 2008; Casella et al., 2019; Kamminga et al., 2006), obtained from the Molecular Signature Database (MSigDB, https://www.broadinstitute.org/gsea/msigdb/msigdb_index.html), as well as a custom signature of genes highly associated with senescent cells (*Cdkn2a, Cdkn1a, Cdkn2d, Casp8, Il1β, Cxcl8, Glb1, Serpine1*). Default parameters were used. Inclusion gene set size was set to a minimum of 5, and the phenotype was permutated 1,000 times.

#### Analysis of scRNA-sequencing data

We accessed the open access data from Van Hove et al. (2019), deposited in https://www.brainimmuneatlas.org/. Details about sample processing, sequencing, and initial QC can be found in the original paper^28^. We accessed the gene-cell count matrix and cell annotation matrix data from 16-month-old APP/PS1 and WT mice, and used Seurat (v3.2.2)^49,50^ for all analyses. A Seurat object was created with default settings, after which a gene list (*Sall1, Gpr34, Tmem119, Hexb, P2ry12,* and *Cx3cr1*) was used to enrich for microglia from the native Cd45^+^ population. Of all cells with a score greater than 0, author-annotated original clusters of microglia and DAM were selected for QC.

As QC thresholds we choose to adopt a 3 × Mean Absolute Deviation (MAD) range for outlier cut-off, as reported previously (Kracht et al., 2020). We determined outliers across four parameters: nCount_RNA, nFeature_RNA, percent.mt, percent.rb. From the initial 6,944 total cells, 2,813 high-quality microglial cells remained after QC.

The filtered matrix was normalized with the NormalizeData-function, using “mean.var.plot” as the selection method. 272 variable genes were identified. We then choose to scale and regress the dataset with ScaleData, regressing for nCount_RNA, nFeature_RNA, percent.mt and IEG-score. For the IEG-score, we made use of the gene list provided by Van Hove et al. (2019), to effectively minimize the effect of dissociation-induced artifacts.

Clustering was performed in a semi-supervised manner, selecting 11 principal components after ‘Jackstraw’ analysis, after which ‘clustree’ guided our selection for a resolution of 0.4. We annotated the resulting microglial clusters by the sample of origin (WT or APP/PS1), as well as enrichment for a DAM signature (*Cst7, Csf1, Lpl, Apoe, Spp1, Cd74, Itgax*) and a custom senescence signature (*Cdkn2a, Cdkn1a, Cdkn2d, Casp8, Il1b, Glb1, Serpine1*). Data was visualized using the DimPlot, FeaturePlot, VlnPlot and DoHeatmap functions, and MAST was used for differential expression analysis (Finak et al., 2015). Quantile ranges for cells positive in senescence score were determined, allowing us to categorize and visualize cells as negative, low, medium and high across conditions.

### Quantification and statistical analysis

#### Image analysis

Images of mouse sections were obtained using a Leica DM4B microscope at X20 magnification and the Olympus VS110 slide scanner for human sections at X40 magnification. When required, we employed the Leica SP8 confocal system. Mouse IHC cell counts were conducted manually (n = *9; 20x fields/mouse*) in the parietal, auditory and entorhinal cortex and averaged. Data were represented as number of positive cells/mm^2^. Human temporal cortex cell counts focused on the gray matter (n = *5-7 brains/group; 10 20xfields/case*). Plaque association analysis was performed using an adapted version of the Sholl analysis, modified from Frautschy et al. (1998). Briefly, we visualized Aβ plaques with congo red staining, tracing concentric circles starting from the diameter of each individual plaque and setting radius step size at 20 μm. Based on our previously published data (Olmos-Alonso et al., 2016), we set a radius of 160 μm to define plaque-association of microglia. Cell density (cells/mm^2^) contained within each circle was quantified, considering that cells falling at the interphase of two areas were counted as belonging to the section containing > 50% of the cell. The quantification of the intensity of signal (i.e., synaptophysin and PSD95) was performed after immunofluorescence and confocal imaging, and presented as %stained area. Amyloid plaque load (6E10) and LAMP1 staining was analyzed by counting density of plaques and area covered (intensity measured by percentage of area). All image analysis was completed using ImageJ, utilizing the color deconvolution plug-in for double brightfield IHCs.

For the morphological analysis of human P16^+^ microglia, gray matter P16 positive and P16 negative microglia were analyzed for differences in morphology using Fiji. 367 cells were analyzed in 7 age-matched AD cases against 7 controls. Soma size and cell surface area were measured manually and using the wand function.

#### Statistical analysis

Data were expressed as mean ± standard error of the mean (SEM) and analyzed with the GraphPad Prism 8 software. For datasets with two or more variables normality and homoscedasticity assumptions were reached, validating the application of the two-way ANOVA, followed by the Tukey post hoc test for multiple comparisons. Human datasets were analyzed using a two-tailed Fisher *t* -test. Differences were considered significant for p < 0.05. Specific details can be found in the figure legends.

## Supplemental information

Document S1. Table S1 and Figures S1–S6

Document S2. Article plus supplemental information
