# Supplementary material for: Replicative senescence dictates the emergence of disease-associated microglia and contributes to Aβ pathology
Source: Cell Rep. 2021 Jun 8;35(10):109228. doi: 10.1016/j.celrep.2021.109228 (PMC8206957; doi:10.1016/j.celrep.2021.109228)
Supplement: Document S1. Table S1 and Figures S1–S6 [file mmc1.pdf]

Cell Reports, Volume *35*

Supplemental information

Replicative senescence dictates the emergence of disease-associated microglia and contributes to Aβ pathology

Yanling Hu, Gemma L. Fryatt, Mohammadmersad Ghorbani, Juliane Obst, David A. Menassa, Maria Martin-Estebane, Tim A.O. Muntslag, Adrian Olmos-Alonso, Monica Guerrero-Carrasco, Daniel Thomas, Mark S. Cragg, and Diego Gomez-Nicola

Replicative senescence dictates the emergence of disease-associated microglia and contributes to Aβ pathology

Yanling Hu, Gemma L. Fryatt, Mohammadmersad Ghorbani, Juliane Obst, David A. Menassa, Maria Martin-Estebane, Tim A. O. Muntslag, Adrian Olmos-Alonso, Monica Guerrero-Carrasco, Daniel Thomas, Mark S. Cragg, Diego Gomez-Nicola

SUPPLEMENTAL FIGURES

Case	Brain Bank ID	Group	Sex	Age	Braak stage	Cause of Death (COD)
SD003/13	BBN_10591	AD	M	86	VI	1a-Congestive cardiac failure<br>2-Alzheimer's Dementia
SD018/13	BBN_15810	AD	F	73	VI	N/A
SD026/13	BBN_15258	AD	M	65	VI	N/A
SD033/13	BBN_15255	AD	F	36	VI	N/A
SD039/13	BBN_19602	AD	M	86	VI	1a-Intracerebral Haemorrhage 1b-Essential (primary) hypertension<br>2 Vascular dementia
SD062/13	BBN_19595	AD	M	87	VI	1a-Intracerebral haemorrhage
SD002/14	BBN_19994	AD	F	87	VI	1a-Other specified respiratory disorder<br>2-Dementia, unspecified
SD025/10	BBN_2522	NDC	M	75	II	1a-Ischaemic heart disease<br>1b-Coronary atherosclerosis
SD014/13	BBN_14395	NDC	F	74	N/A	1a-Pulmonary thromboembolism
SD010/09	BBN_2469	NDC	M	66	N/A	1a-Haemopericardium<br>1b-Ruptured myocardial infarct<br>1c-Coronary artery thrombosis<br>1d-Coronary artery atherosclerosis<br>2-Hypertensive heart disease
SD001/11	BBN_2540	NDC	M	74	N/A	1a-Pulmonary thromboembolism<br>1b-Deep vein thrombosis
SD023/10	BBN_2520	NDC	M	75	0	1a-Ischaemic heart disease<br>1b-Coronary artery atherosclerosis
SD003/14	BBN_20122	NDC	M	59	N/A	1a-Myocardial infarction<br>1b-Coronary artery atherosclerosis<br>2-Type 1 Diabetes Mellitus
SD030/12	BBN_4169	NDC	F	71	N/A	1a-Ischaemic and hypertensive heart disease

Supplemental Table 1. Pathology of the post-mortem samples from AD or age-matched non-demented controls (NDC) used in the histological study (related to Methods and Figure 4). Data provided by the National CJD Surveillance Unit Brain Bank (Edinburgh, UK).

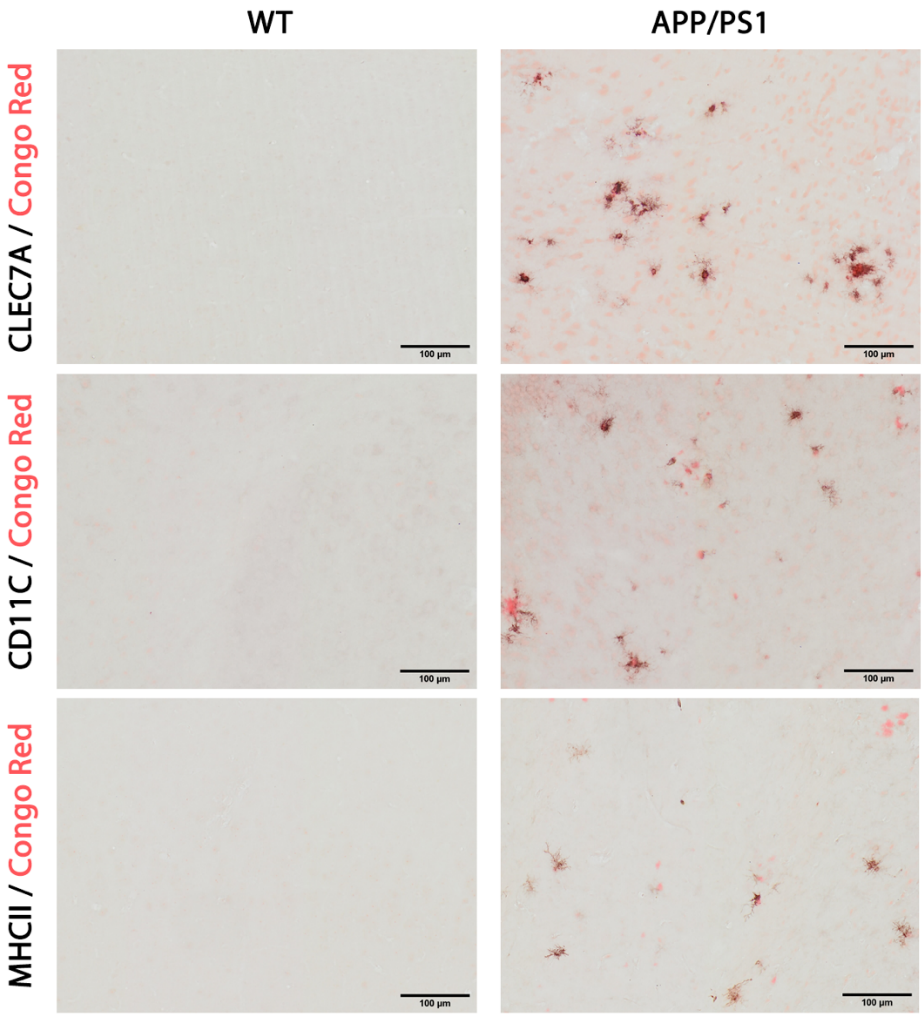

Figure S1. Representative images of the IHC for DAM markers (CLEC7A, CD11C, MHCII), in APP/PS1 mice and WT littermate controls (related to Figure 1). Aβ plaques labelled with Congo Red. All samples collected from cerebral cortex. Scale bar 100μm.

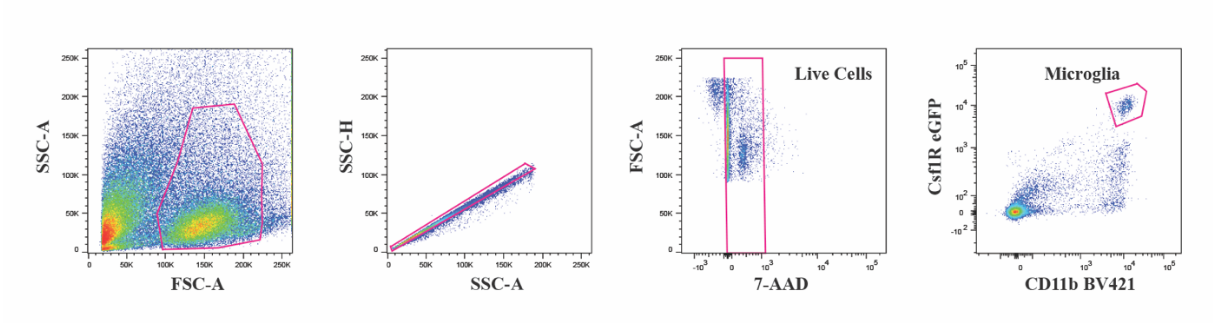

Figure S2. Representative example of the gating strategy for analysing and sorting microglia (related to Figures 1, 2, 3). Cells from the target population are gated as singlets and live cells, selecting microglia from the CSF1R eGFP^{+} CD11B^{+} gate.

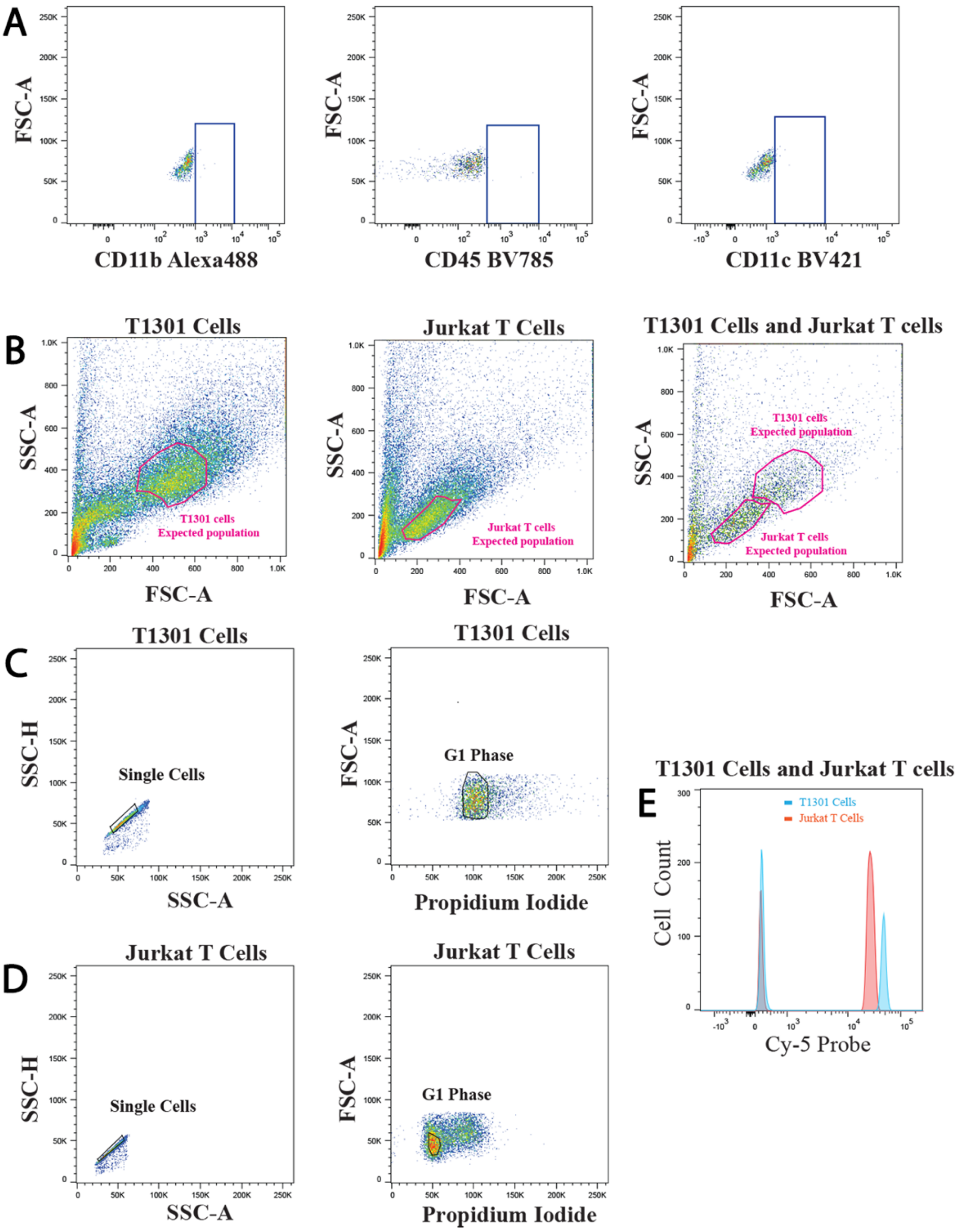

Figure S3. Representative example of the Flow-FISH gating strategy and data analysis for T1301 cells and Jurkat T cells (related to Figure 2).

(A) Unstained negative control sample for each individual fluorescent dye used in Flow-FISH, to determine positive staining for the samples. (B) Identification of T1301 and Jurkat T cell populations in a SSC/FSC dot plot. (C, D) Gating of T1301 cells (C) and Jurkat T cells (D) by selection of singlets in SSC-H/SSC-A followed by selection of haploid cells in FSC-A/PI. (E) Cy-5 (telomere probe) fluorescence histograms (median fluorescence intensity; MFI) of the gated T1301 and Jurkat T cell populations, showing unstained controls (left) and stained samples (right).

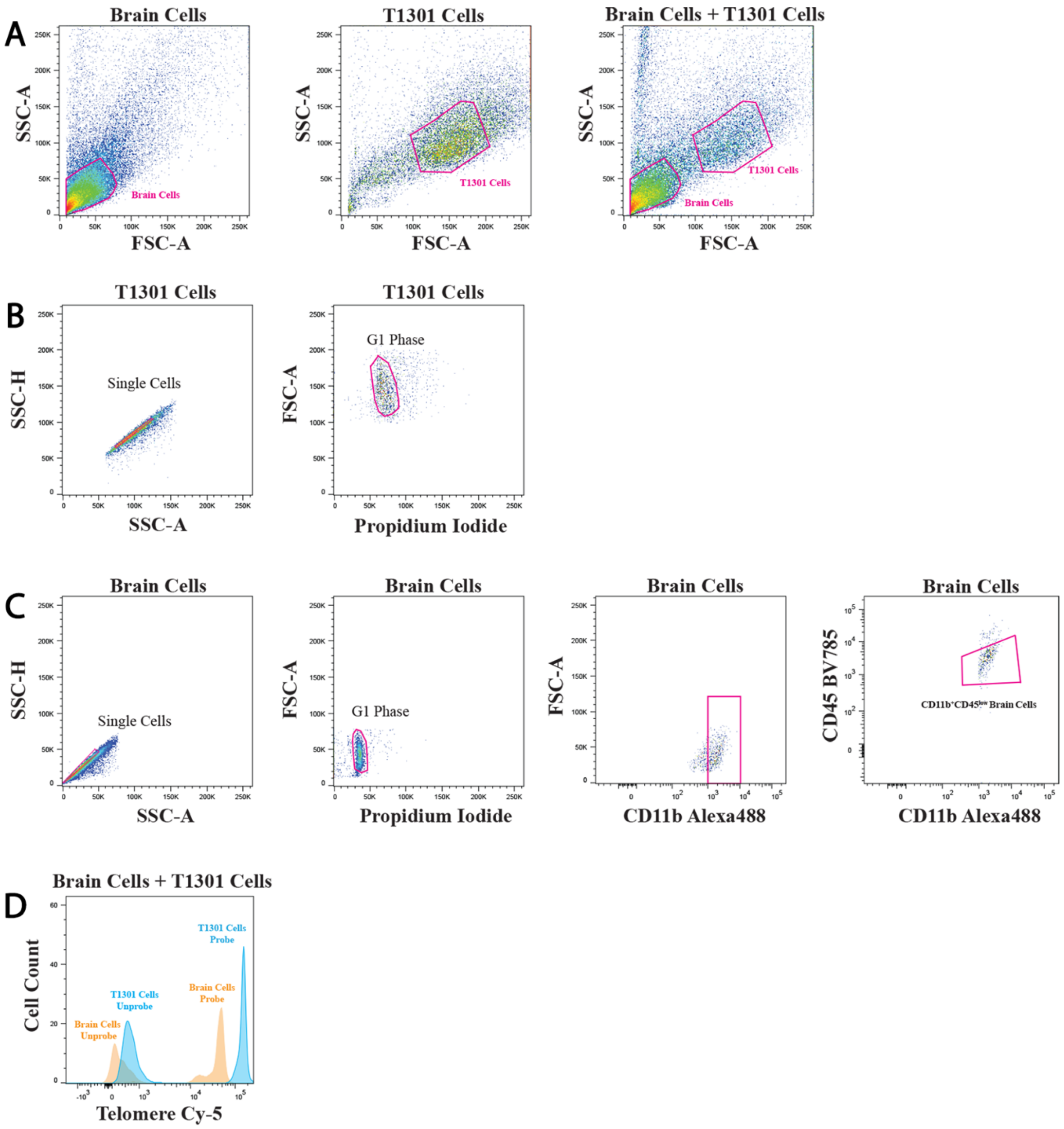

Figure S4. Representative example of Flow-FISH gating strategy and data analysis of microglia mixed with T1301 cells (related to Figure 2).

(A) Identification of T1301 cells and brain cells in a SSC/FSC dot plot. (B) Gating of T1301 cells as singlets in SSC-H/SSC-A followed by selection of haploid cells in FSC-A/PI. (C) Gating of brain cells as singlets in SSC-H/SSC-A followed by selection of haploid cells in FSC-A/PI, further gating microglia as $CD11b^{+}CD45^{low}$. (D) Cy-5 (telomere probe) fluorescence histograms (median fluorescence intensity; MFI) of the gated T1301 and brain cell populations, showing unstained controls (left) and stained samples (right).

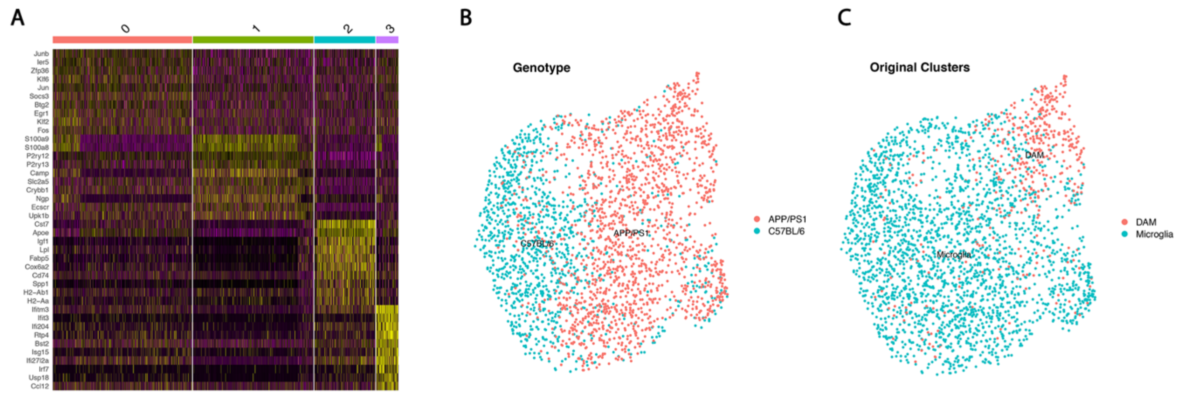

Figure S5. Analysis of the single-cell dataset from Van-Hove et al., 2019[28] (related to Figure 3). (A) Heat map of the key genes characterising the 4 clusters identified in the dataset. (B) UMAP plot identifying genotype of origin (C57BL/6 or APP/PS1), as annotated in Van-Hove et al., 2019[28]. (C) UMAP plot identifying clusters (DAM and microglia), originally annotated in Van-Hove et al., 2019[28].

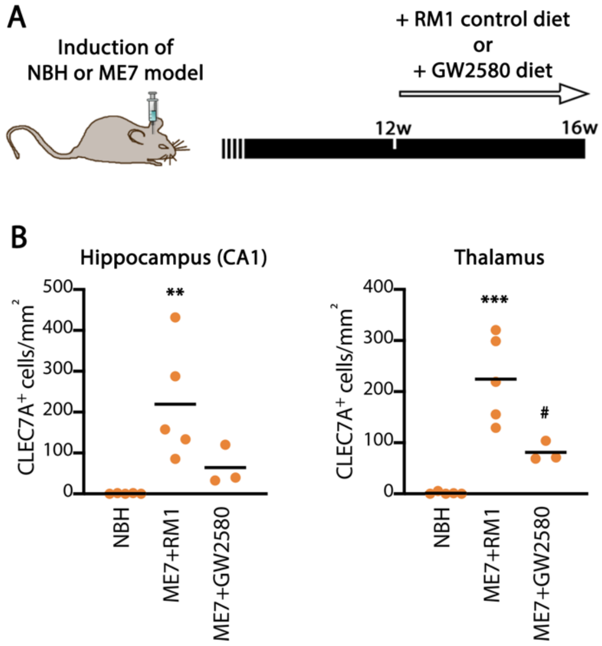

Figure S6. Inhibition of CSF1R prevents the onset of DAM in a model of prion disease (related to Figure 5).

(A) Graphical representation of the experimental model of prion disease (ME7) and treatments from 12 weeks post-induction until 16 weeks post-induction. (B) Density of DAM, identified as CLEC7A$^+$ cells, in control (NBH; normal brain homogenate) and prion-diseased (ME7) mice, after treatment with control (RM1) or GW2580-containing diet, analysed by IHC. IHC data collected from the hippocampus (CA1) or thalamus. Data shown in represented as mean±SEM. Statistical differences: **p<0.01, ***p<0.001, *vs* NBH; $^{\#}$p<0.05, *vs* ME7+RM1 group. Data were analysed with a one-way ANOVA.
